# The risk of Type 1 diabetes in children born after ART: a Nordic cohort study from the CoNARTaS group

**DOI:** 10.1093/hropen/hoae021

**Published:** 2024-04-10

**Authors:** Frederik Kyhl, Anne Lærke Spangmose, Mika Gissler, Kristiina Rönö, Kjersti Westvik-Johari, Anna-Karina Aaris Henningsen, Christina Bergh, Ulla-Britt Wennerholm, Signe Opdahl, Julie Forman, Jannet Svensson, Tine Clausen, Ditte Vassard, Anja Pinborg

**Affiliations:** Fertility Clinic, Department of Gynaecology, Fertility and Obstetrics, Copenhagen University Hospital, Rigshospitalet, Copenhagen, Denmark; Fertility Clinic, Department of Gynaecology, Fertility and Obstetrics, Copenhagen University Hospital, Rigshospitalet, Copenhagen, Denmark; Department of Knowledge Brokers, Finnish Institute for Health and Welfare, Helsinki, Finland; Department of Molecular Medicine and Surgery, Karolinska Institute, Stockholm, Sweden; Department of Obstetrics and Gynecology, Helsinki University Hospital and University of Helsinki, Helsinki, Finland; Department of Fertility, Women and Children’s Centre, St Olavs Hospital, Trondheim, Norway; Faculty of Medicine and Health Sciences, Department of Public Health and Nursing, Norwegian University of Science and Technology, Trondheim, Norway; Fertility Clinic, Department of Gynaecology, Fertility and Obstetrics, Copenhagen University Hospital, Rigshospitalet, Copenhagen, Denmark; Department of Obstetrics and Gynecology, Institute of Clinical Sciences, Sahlgrenska Academy, Gothenburg University, Region Västra Götaland, Sahlgrenska University Hospital, Gothenburg, Sweden; Department of Obstetrics and Gynecology, Institute of Clinical Sciences, Sahlgrenska Academy, Gothenburg University, Region Västra Götaland, Sahlgrenska University Hospital, Gothenburg, Sweden; Faculty of Medicine and Health Sciences, Department of Public Health and Nursing, Norwegian University of Science and Technology, Trondheim, Norway; Department of Public Health, Section of Biostatistics, University of Copenhagen, Copenhagen, Denmark; Department of Paediatric and Adolescents, Copenhagen University Hospital, Herlev & Gentofte, Denmark; Department of Clinical Medicine, University of Copenhagen, Copenhagen, Denmark; Department of Clinical Research, Steno Diabetes Center Copenhagen, Herlev, Denmark; Department of Obstetrics, Copenhagen University Hospital, Rigshospitalet, Copenhagen, Denmark; Fertility Clinic, Department of Gynaecology, Fertility and Obstetrics, Copenhagen University Hospital, Rigshospitalet, Copenhagen, Denmark; Fertility Clinic, Department of Gynaecology, Fertility and Obstetrics, Copenhagen University Hospital, Rigshospitalet, Copenhagen, Denmark; Department of Clinical Medicine, University of Copenhagen, Copenhagen, Denmark

**Keywords:** assisted reproductive technology, ART, Type 1 diabetes, frozen embryo transfer, long-term outcome, registry-based study

## Abstract

**STUDY QUESTION:**

Do children born after ART have a higher risk of developing Type 1 diabetes (DM1) than children conceived without ART?

**SUMMARY ANSWER:**

The risk of DM1 was similar for children conceived with and without ART, and there were no clear differences in risk according to method of fertility treatment.

**WHAT IS KNOWN ALREADY:**

ART is associated with a higher risk of adverse perinatal outcomes, and the risk depends on the method of ART. The Developmental Origins of Health and Disease theory proposes that prenatal stress can provoke changes in endocrine processes which impact health later in life.

**STUDY DESIGN, SIZE, DURATION:**

A Nordic register-based cohort study was carried out, including all children born in Denmark (birth years 1994–2014), Finland (1990–2014), and Norway (1984–2015). The study included 76 184 liveborn singletons born after ART and 4 403 419 born without ART. Median follow-up was 8.3 and 13.7 years in the ART and non-ART group, respectively.

**PARTICIPANTS/MATERIALS, SETTING, METHODS:**

The cohort, initiated by the Committee of Nordic Assisted Reproductive Technology and Safety (CoNARTaS), was established by linking national registry data from the medical birth registries and national patient registries available in the Nordic countries. We performed multivariable logistic regression analyses for the birth year intervals 1984–1990, 1991–1995, 1996–2000, 2001–2005, 2006–2010, and 2011–2015, while adjusting for year of birth within each interval, sex of the child, parity, maternal age, maternal diabetes, and maternal smoking during pregnancy as potential confounders.

**MAIN RESULTS AND THE ROLE OF CHANCE:**

During follow-up, 259 (3.4‰) children born after ART were diagnosed with DM1, while this was the case for 22 209 (5.0‰) born without ART, corresponding to an adjusted odds ratio of 0.98 (95% CI: 0.861.11). Within the different birth year intervals, no significant difference in risk of DM1 between the two groups was found, except for the youngest cohort of children born 2011–2015 where ART was associated with a higher risk of DM1. We found no significant differences in risk of DM1 when comparing children born after IVF versus ICSI or fresh versus frozen embryo transfer, but with only few cases in each group.

**LIMITATIONS, REASONS FOR CAUTION:**

The main limitation of the study is the relatively short follow-up time. The incidence rate of DM1 peaks during ages 10–14 years, hence a longer follow-up would benefit all analyses and, in particular, the subgroup analyses.

**WIDER IMPLICATIONS OF THE FINDINGS:**

Overall, our findings are reassuring especially considering the concomitantly increasing number of children born from ART and the increasing incidence of DM1 globally.

**STUDY FUNDING/COMPETING INTEREST(S):**

This Nordic registry study has been supported by the Nordic Trial Alliance/NORDFORSK and Rigshospitalets Research Foundation. The funding sources had no role in study design; in the collection, analysis, and interpretation of data; in the writing of the report; and in the decision to submit the article for publication. None of the authors has any conflicts of interest to declare regarding this study.

**TRIAL REGISTRATION NUMBER:**

ISRCTN11780826.

WHAT DOES THIS MEAN FOR PATIENTS? This study investigates the risk of Type 1 diabetes in children born from assisted reproductive technology (ART). The Developmental Origins of Health and Disease theory proposes that stress of the pregnant mother and foetus can impact the health of the child later in life. Previous studies have found that children conceived by ART with frozen embryo transfer had an increased risk of Type 1 diabetes. Through the use of large registry data in Denmark, Norway, and Finland, we found no increased risk of Type 1 diabetes after ART conception overall, or after specific methods such as IVF, ICSI, and frozen embryo transfer.

## Introduction

The number of children born after ART is increasing globally. In 2017, 1.96 million ART cycles and more than 330 000 births were registered worldwide (Adamson, 2021). The Nordic countries are among those with the highest rates of ART in the world (Gliozheni *et al.*, 2023) and in recent years, one in ten births in Denmark is the result of some form of fertility treatment (Lemmen *et al.*, 2022). ART with fresh embryo transfer (ET) is associated with higher rates of preterm birth, low birthweight, and being born small for gestational age (SGA) (Henningsen *et al.*, 2011; Pandey *et al.*, 2012; Pinborg *et al.*, 2013). Children born preterm are also at higher risk of Type 1 diabetes (DM1) (Li *et al.*, 2014). Conversely, children born after frozen embryo transfer (FET) have an increased risk of being born large for gestational age (LGA) and with high birthweight, compared to both fresh ET and natural conception (Berntsen and Pinborg, 2018), which is associated with an increased risk of DM1 (Harder *et al.*, 2009; Cardwell *et al.*, 2010; Magnusson *et al.*, 2021). The Developmental Origins of Health and Disease theory hypothesizes that prenatal stress provokes changes in endocrine processes which impact health later in life (Barker, 2007; Sullivan *et al.*, 2008). Vrooman and Bartolomei (2017) hypothesized that ART-induced epigenetic modulation at the early embryonic and foetal stages may affect the long-term health of the children.

A nationwide registry study on 3 138 540 children born in Sweden showed no association between ART and DM1 when compared to children conceived without ART, but a higher risk of DM1 in children born after FET compared to fresh ET and children conceived without ART (Norrman *et al.*, 2020). Two Danish studies partly overlapping with our cohort had reassuring results. A cohort study by Kettner *et al.* (2016), including 565 116 children with 14 985 born after ART, found no increased risk of DM1 in ART-conceived children, but did interestingly find an increased risk of DM1 comparing children born from IUI and non-ART (Kettner *et al.*, 2016). Hargreave *et al.* (2016) compared 110 393 children of mothers with a diagnosis of infertility to an unexposed group of 1 550 519 children born between 1987 and 2010 and found no difference in risk of DM1 (Hargreave *et al.*, 2016). Klemetti *et al.* (2006), a Finnish study, also partly overlapping with our cohort, did not find a difference in the risk of DM1 up to age 4 years comparing 4559 children born after ART compared to 190 398 born after non-ART conception. However, they did find a higher prevalence of DM1 in the children conceived by ART. In the total ART group, 0.9/1000 were diagnosed with DM1 compared to 0.5/1000 in the non-ART group (Klemetti *et al.*, 2006).

Despite substantial improvement in the treatment of DM1 over the years, the condition is still associated with increased morbidity and mortality (Maahs *et al.*, 2010; Gregory *et al.*, 2022). The incidence of DM1 is increasing globally, as well as in the Nordic countries (Atkinson *et al.*, 2014; Gregory *et al.*, 2022), with the Finnish population having the highest observed incidence rate of DM1 worldwide (>60 cases per 100 000 person years) (Maahs *et al.*, 2010). Although several risk factors associated with DM1 are well known, including male sex, non-Hispanic white ethnicity, higher maternal age at delivery and parental DM1 (Maahs *et al.*, 2010), the causes of the increasing DM1 incidence are not yet fully understood.

The risk of DM1 has been increasing concomitantly with the rising use of ART treatment. Thus, this study aimed to estimate the risk of DM1 in children conceived after ART compared with children conceived without ART. We further investigated whether various methods of ART were associated with the risk of DM1. This is the largest cohort study on ART and the association to DM1 to date, and includes nationwide populations from three different Nordic countries.

## Materials and methods

### Data sources

This is a registry-based cohort study using data from the Committee of Nordic ART and Safety (CoNARTaS) cohort (Opdahl *et al.*, 2020). In short, the CoNARTaS cohort contains information from the national Medical Birth Registries (MBRs) in Denmark, Finland, Norway, and Sweden, where maternal, obstetric, and perinatal outcomes are registered. Data from the national MBRs were linked to data from National Patient Registries (NPRs), containing information on individually registered inpatient care (all years in Finland and Denmark, and Norway since 2008) and outpatient visits in public hospitals (Finland since 1998) or in public and private health clinics (Denmark since 1995 and Norway since 2008). The DM1 registration in the Danish NPR has been shown to have a sensitivity of 96% and a specificity of 100% (Svensson *et al.*, 2007). Date of death was available in the Cause of Death Registry, while data on emigration was available in the population registries. Emigration dates were not available in Finland. Linkage of health care data is possible in the Nordic countries as all residents receive a unique national identity number at birth or immigration, which is used in contact with health care services and in reporting to the national health registries (Laugesen *et al.*, 2021).

### Study population

**Figure 1.**
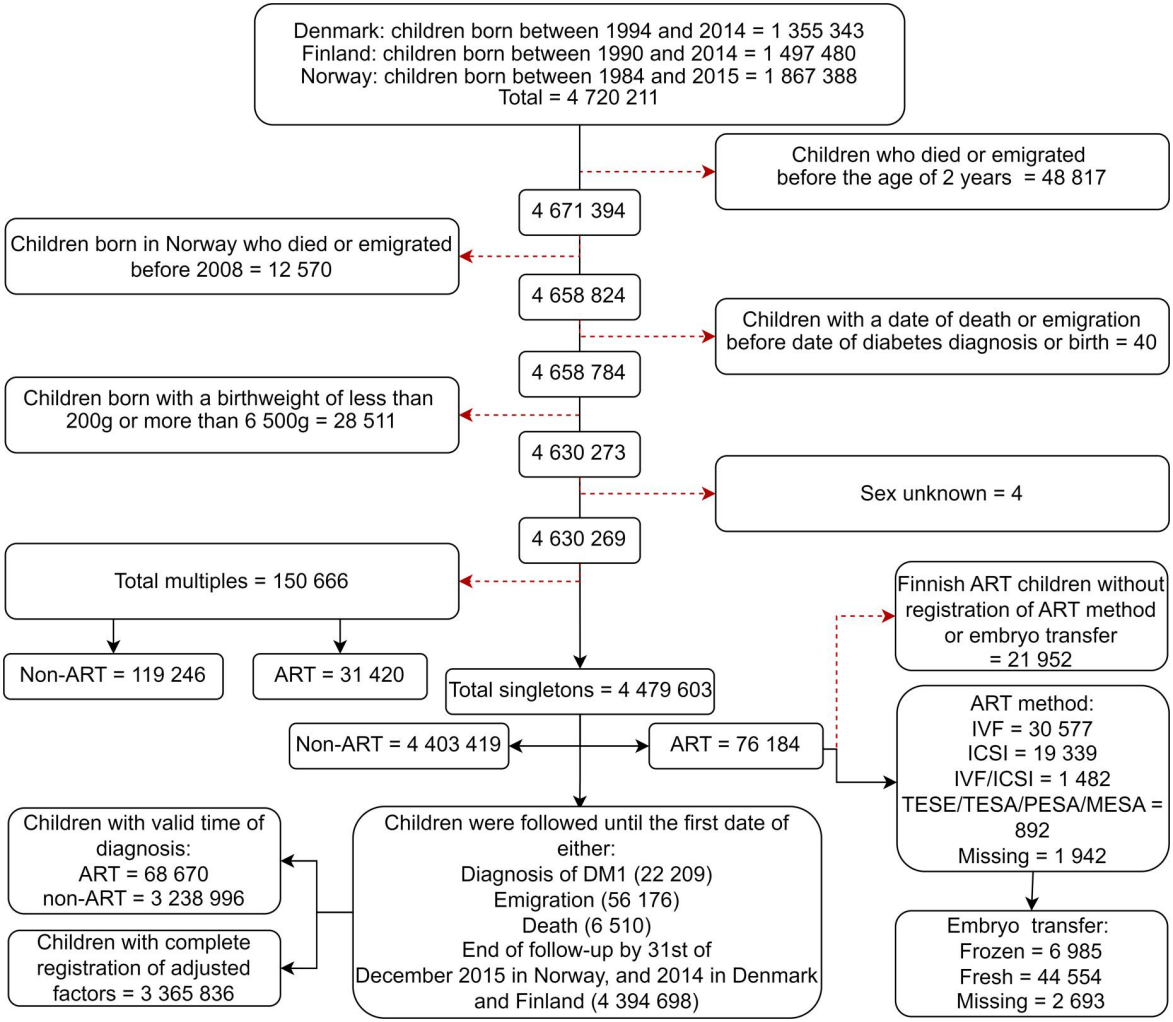
**Flow diagram detailing inclusion and exclusion criteria in a Nordic cohort study of the risk of Type 1 diabetes in children born after ART.** DM1, Type 1 diabetes; MESA, microsurgical epididymal sperm aspiration; PESA, percutaneous epididymal sperm extraction; TESA, testicular sperm aspiration; TESE, testicular sperm extraction.

We included all liveborn children born in Denmark (birth years 1994–2014), Finland (1990–2014), and Norway (1984–2015). We divided the cohort into singletons and multiples. Our main analysis included 76 184 ART-conceived singletons and 4 403 419 singletons born after non-ART. For the subgroup analysis of multiples, we included 31 420 children born from ART and 119 246 from non-ART. Exclusion criteria were children who died or emigrated before 2 years of age, children with missing or outlying birthweight of <200 g or higher than 6500 g, and children with unknown sex. Owing to concerns about data validity, children registered as dead or emigrated before a registered diagnosis of DM1 were excluded (Fig. 1). Children from Norway who died or emigrated before 2008 were also excluded as the national identity number was not introduced in the NPR until 2008, and data on DM1 diagnosis could not be linked for specialist healthcare contacts before that. Since a study on the risk of DM1 in ART-conceived children based on Swedish CoNARTaS data has already been performed, we did not include data from Sweden (Norrman *et al.*, 2020).

Children born after IUI were included in the non-ART cohort for all three countries. Comparing children born after IVF and ICSI, we excluded children with unknown fertilization methods and children conceived using non-ejaculated sperm. For analyses comparing FET and fresh ET, we excluded children registered as conceived using non-ejaculated sperm or with a missing registration of whether the embryo was fresh or frozen. Data from Finland were only included in the comparison of ART versus non-ART, as more detailed information on IVF, ICSI, and FET cycles was not available.

### Outcome

DM1 was defined as having an International Classification of Diseases and Related Health Problems (ICD) 8th (ICD-8) or 9th revision (ICD-9) *code 250* (Finland 1990–1995) or 10th revision (ICD-10) *code E10*.

### Covariates

The following covariates were included: year of birth (defined intervals and continuous), sex of the child (male/female), parity (0, 1, or ≥2), maternal age (continuous), maternal diabetes (yes/no), and maternal smoking during pregnancy (yes/no). Maternal smoking during pregnancy was not registered in Norway before 1999. Maternal DM1 was defined as having an ICD-8 or ICD-9 *code 250* (Finland 1986–1995) or ICD-10 *code E10* at any time during follow-up. We had no data on paternal DM1 diagnoses.

SGA and LGA were defined as < −2SD and > +2SD from the expected sex-specific birthweight for the given gestational age, respectively (Maršál *et al.*, 1996). Maternal education was defined as the last educational level registered within the study period, with the categories being low, middle, and high. Data on education were not available from Norway.

### Statistical analyses

In our primary analysis, we compared the risk of DM1 in ART versus non-ART using multiple logistic regression, stratified on birth-year intervals 1984–1990, 1991–1995, 1996–2000, 2001–2005, 2006–2010, and 2011–2015, to study time trends and to account for the various follow-up times. Potential confounders were defined as factors that could influence both the need for ART treatment and the risk of DM1. Data on the following confounders were available in the registries: year of birth, sex of the child, parity, maternal age, maternal diabetes, and maternal smoking during pregnancy. Among these, adjustment for year of birth had the strongest impact on the estimates and was thus included in all models. We conducted the analyses for each time period according to the following models: Model 1: Adjustment for year of birth within each interval only, Model 2: Adjustment for year of birth, sex of the child, parity, maternal age, and maternal diabetes, and Model 3: Adjustment according to Model 2 plus maternal smoking during pregnancy. Model 2 was set as the primary model because the proportion of missing values for maternal smoking during pregnancy, included only in Model 3, was high.

To investigate if associations differed by the type of ART treatment, we repeated Model 2 comparing the risk of DM1 between: children born after ICSI versus IVF; FET versus fresh ET; FET versus children conceived without ART; and finally fresh ET versus children conceived without ART.

Stratified subgroup analyses were made to compare ART and non-ART by country and by sex. Further subgroup analyses were restricted to children born at term and with birthweight appropriate for gestational age. We performed analyses restricted by gestational age and birthweight because children born preterm have a higher risk of DM1 (Li *et al.*, 2014), as do children born LGA and with a high birthweight (Harder *et al.*, 2009; Cardwell *et al.*, 2010; Magnusson *et al.*, 2021). To investigate how exclusion criteria and analytical approaches affected the results, we conducted sensitivity analyses based on Model 2, on the following samples: multiples, and children with at least 2 years of follow-up. The sample of multiple included all multiples selected according to the criteria in the singletons study population (Fig. 1).

Finally, we repeated the analysis on children born from ART versus non-ART in a subpopulation restricted to children where the child’s age at the time of diagnosis was available. This subpopulation included 78.8% of the total study population of singletons, but excluded individuals who were born in Norway prior to 2006, as their age at diagnosis could not be accurately determined. In this subsample with available age at diagnosis, we performed Cox regression analyses taking differences in follow-up and age at diagnosis into account. Time at risk was defined as the time from birth to the time of DM1 diagnosis or censoring at emigration, death, or end of follow-up, which was 31 December 2014 for Denmark and Finland and 31 December 2015 for Norway. Additionally, because the full cohort did not contain a valid time of diagnosis for maternal DM1 among all mothers, we repeated the adjustment for maternal DM1 in this subpopulation, defining maternal DM1 as pre-gestational DM1 only.

Associations were considered as statistically significant if the 95% CI did not contain an odds ratio (OR) (or hazard ratio, HR) of 1. All statistical analyses were performed using SAS software version 9.4 (SAS Institute Inc 2013. SAS/ACCESS^®^ 9.4. SAS Institute Inc. Cary, NC, USA).

### Ethical approval

The study was approved by the Data Protection Agencies and registry-keeping authorities in Denmark and Finland. In Norway, ethical approval was given by the Regional Committee for Medical and Health Research Ethics (REK-Nord, 2010/1909).

## Results

The mean birthweight was 3441 g (SD 606 g) in the ART group and 3547 g (SD 553 g) in the non-ART group. In the ART group, 7.8% were born pre-term, 4.7% were born SGA, and 4.3% were LGA compared to 4.6%, 3.7%, and 4.8% in the non-ART group, respectively. The mothers had a mean age at birth of 33.8 and 29.6 years, a proportion of 14.8% and 8.5% with a high educational level, and a proportion of 6.7% and 12.0% smoked during pregnancy in the ART and non-ART population, respectively. In Denmark, the proportion of children born after ART of the total population was 2.4% (30 368 of 1 255 171). In Finland, the proportion of children conceived from ART was 1.5% and in Norway, it was 1.3% (Table 1).

**Table 1.** Characteristics of singletons born after ART and non-ART, and their mothers.

	ART	Non-ART	All
	N = 76 184	N = 4 403 419	N = 4 479 603
**Child characteristics**			
Child’s sex (female)—no. (%)	37 269 (48.9)	2 146 158 (48.7)	2 183 427 (48.7)
Birthweight (g)—mean (SD)	3441 (606)	3547 (553)	3545 (554)
Birthweight 1500–2499 g—no. (%)	3455 (4.5)	117 809 (2.7)	121 264 (2.7)
Very low birthweight <1500 g—no. (%)	826 (1.1)	20 849 (0.5)	21 675 (0.5)
High birthweight 4000–4499 g—no. (%)	9856 (12.9)	700 186 (15.9)	710 042 (15.9)
Very high birthweight ≥4500 g—no. (%)	2175 (2.9)	158 163 (3.6)	160 338 (3.6)
Gestational age (days)—median (IQR)	279 (271–286)	281 (273–287)	280 (273–287)
Preterm birth <259 days—no. (%)	5943 (7.8)	201 539 (4.6)	207 482 (4.6)
Very preterm birth <224 days—no. (%)	971 (1.3)	25 863 (0.6)	26 834 (0.6)
Missing (%)	248 (0.3)	90 312 (2.0)	90 560 (2.0)
Small for gestational age—no. (%) ^∫^	3544 (4.7)	161 117 (3.7)	164 661 (3.7)
Large for gestational age—no. (%)^∫^	3270 (4.3)	211 158 (4.8)	214 428 (4.8)
Country of birth—no. (%)			
Denmark	30 368 (39.9)	1 224 803 (27.8)	1 255 171 (28.0)
Finland	21 952 (28.8)	1 423 900 (32.3)	1 445 852 (32.3)
Norway	23 864 (31.3)	1 754 716 (39.8)	1 778 580 (39.7)
Follow-up (years)—median (IQR)	8.3 (4.3–13.9)	13.7 (7.2–20.3)	13.6 (7.1–20.2)
Denmark	8.5 (4.6–13.4)	11.6 (6.4–16.9)	11.6 (6.4–16.9)
Finland	9.6 (4.9–15.6)	13.8 (7.3–20.3)	13.7 (7.3–20.2)
Norway	7.1 (3.4–12.5)	16.0 (7.7–23.7)	15.8 (7.6–23.6)
**Maternal characteristics**			
Maternal age (years)—mean (SD)	33.8 (4.4)	29.6 (5.2)	29.7 (5.2)
Maternal BMI (kg/m^2^)—median (IQR)^θ^	23.1 (21.0–26.2)	23.2 (21.0–26.4)	23.2 (21.0–26.4)
Missing—no. (%)	36 200 (47.5)	2 988 284 (67.9)	3 024 484 (67.5)
Maternal Type 1 diabetes—no. (%)^⁑^	535 (0.7)	32 170 (0.7)	32 705 (0.7)
Maternal educational level years—no. (%)^¤^			
Low	21 955 (28.8)	1 319 862 (30.0)	1 341 817 (30.0)
Middle	17 576 (23.1)	773 919 (17.6)	791 495 (17.7)
High	11 283 (14.8)	372 848 (8.5)	365 852 (8.6)
Missing	25 370 (33.3)	1 936 790 (44.0)	1 962 160 (43.8)
Maternal smoking during pregnancy—no. (%)	5082 (6.7)	528 680 (12.0)	533 762 (12.1)
Missing	7729 (10.1)	1 101 091 (25.0)	1 108 820 (24.8)
Caesarean section—no. (%)	20 075 (26.4)	660 911 (15.0)	680 986 (15.2)
Nulliparous—no. (%)	50 166 (65.8)	1 824 402 (41.4)	1 874 568 (41.8)
Missing (%)	287 (0.4)	6098 (0.1)	6385 (0.1)

IQR, interquartile range.

∫ Small and large for gestational age were defined as < −2SD and > +2SD from the expected sex-specific birth weight for the given gestational age (Maršál *et al.*, 1996).

θ Maternal BMI was available since 2004 for Denmark and Finland and since 2007 for Norway.

⁑ Maternal diabetes was registered as ICD-8 and ICD-9 diagnoses in 13.5% of the cases, and no distinction between Types 1 and 2 diabetes could be made.

¤ There were no available data on maternal educational level from Norway.

### Risk of DM1 in the total population

Median follow-up was 8.3 years (IQR 4.3–13.9) and 13.7 years (IQR 7.2–20.3) in the ART and non-ART group, respectively. During follow-up, 259 (3.4 ‰) children conceived after ART were diagnosed with DM1, with a corresponding 22 209 (5.0 ‰) diagnosed in the non-ART group. Multivariable regression analyses performed according to all models of adjustment with the total cohort showed no significant difference in the odds of DM1 between ART- and non-ART-conceived children (adjusted OR (aOR) 0.98, 95% CI: 0.86–1.11). In the analyses stratified by birth year categories, no significant differences in odds of DM1 between the two groups were found except for the youngest birth cohort (2011–2015) with an aOR 1.76 (95% CI: 1.10–2.82). Further adjustments gave similar results as in Model 1 (Table 2).

**Table 2.** Multivariable logistic regression analyses for singletons born after ART versus non-ART in the total cohort—risk of Type 1 diabetes during follow-up.

Birth year	ART	Non-ART	**Adj. OR** ^1^	**Adj. OR** ^2^	**Adj. OR** ^3^
	no. (‰)	no. (‰)	(95% CI)	(95% CI)	(95% CI)
1984–1990^⁑^	5/493 (10.0)	3408/429 219 (7.9)	1.24 (0.52–3.00)	1.24 (0.51–3.01)	***
1991–1995	29/3716 (7.8)	5846/726 936 (8.0)	0.99 (0.69–1.43)	0.95 (0.66–1.38)	0.98 (0.63–1.54)
1996–2000	76/11 900 (6.4)	6056/859 872 (7.0)	0.92 (0.73–1.15)	0.89 (0.71–1.13)	0.96 (0.75–1.22)
2001–2005	78/15 794 (4.9)	4153/826 896 (5.0)	0.99 (0.79–1.24)	1.00 (0.80–1.26)	1.00 (0.79–1.26)
2006–2010	52/21 885 (2.4)	2109/854 703 (2.5)	0.99 (0.75–1.30)	1.07 (0.81–1.41)	1.05 (0.79–1.40)
2011–2015	19/22 396 (0.8)	378/705 793 (0.5)	1.62 (1.02–2.58)	1.76 (1.10–2.82)	1.69 (1.02–2.78)
Total	259/76 184 (3.4)	21 950/4 403 419 (5.0)	0.99 (0.87–1.12)	0.98 (0.86–1.11)	1.03 (0.90–1.17)

Associations are presented as adjusted odds ratios (OR). Follow-up is described in Supplementary Table S2.

Adj., adjusted.

1 Adjusted for year of birth.

2 Adjusted for year of birth, sex of the child, parity, maternal age, and maternal diabetes.

3 Adjusted for year of birth, sex of the child, parity, maternal age, maternal diabetes, and maternal smoking during pregnancy.

⁑ For the birth years 1984–1989 and 2015 only data from Norway were included.

*** Unable to perform analysis owing to limited observations.

Supplementary Fig. S1 displays the associations of the included covariates with risk of DM1. The strongest association for developing DM1 was observed for maternal DM1. Male sex and no maternal smoking during pregnancy were also associated with a higher risk of developing DM1.

### Risk of DM1 in the subpopulation

When including children born in Finland and Denmark during the full study period as well as Norwegian children born from 2006 onwards, the age at the end of follow-up and the child’s age at diagnosis remained substantially lower in the ART group compared to the non-ART group. However, within each birth year interval, the age at the end of follow-up was comparable, while the age at diagnosis varied somewhat between the ART and non-ART groups, but with no systematic pattern across the birth year intervals (Supplementary Table S1). In this subpopulation, we found no differences in risk between the ART and non-ART groups, neither in the multivariable logistic nor Cox regression analyses (Supplementary Table S2). Cumulative hazards of DM1 according to child age in this cohort showed that ART-conceived children had a similar age at diagnosis of DM1 compared to children conceived without ART (Supplementary Fig. S2).

### Risk of DM1 after various ART methods

#### ICSI versus IVF

In total, 39 children born after ICSI (2.0‰) and 81 children born after IVF (2.6‰) were diagnosed with DM1 during the observational period. We found similar odds of DM1 in the ICSI versus the IVF group, with an aOR of 1.10 (95% CI: 0.73–1.65) (Table 3).

**Table 3.** Multivariable logistic regression analyses for singletons born after ICSI versus IVF, frozen ET versus fresh ET and non-ART and fresh ET versus non-ART—risk of Type 1 diabetes during follow-up.

	Type 1 diabetes no. (‰)	Median follow-up—years (IQR)	Group versus reference	**Adj. OR** ^1^	**Adj. OR** ^2^	**Adj. OR** ^3^
				(95% CI)	(95% CI)	(95% CI)
**ICSI**	39/19 339 (2.0)	6.6 (3.5–10.6)	**ICSI versus IVF**	1.10 (0.73–1.65)	1.10 (0.73–1.65)	1.03 (0.67–1.60)
**IVF**	81/30 577 (2.6)	9.1 (4.6–15.0)				
**FET**	10/6985 (1.4)	5.3 (2.6–8.9)	**FET versus Fresh ET**	0.82 (0.42–1.57)	0.79 (0.41–1.53)	0.77 (0.37–1.60)
**Fresh ET**	109/44 554 (2.4)	8.4 (4.4–13.4)	**FET versus Non-ART**	0.74 (0.40–1.37)	0.74 (0.40–1.38)	0.86 (0.43–1.72)
**Non-ART**	11 570/2 979 519 (3.9)	13.7 (7.1–20.3)	**Fresh ET versus Non-ART**	1.00 (0.82–1.20)	1.01 (0.84–1.23)	1.12 (0.90–1.40)

Associations are presented as adjusted odds ratios (OR). Data on frozen embryo transfer (FET) and fresh ET were available only for Denmark (35 missing) and Norway (2658 missing).

Adj., adjusted; IQR, interquartile range.

1 Adjusted for year of birth.

2 Adjusted for year of birth, sex of the child, parity, maternal age, and maternal diabetes.

3 Adjusted for year of birth, sex of the child, parity, maternal age, maternal diabetes, and maternal smoking during pregnancy.

#### Frozen versus fresh ET

In total, 10 children born after FET (1.4‰), 109 children born after fresh ET (2.4‰), and 11 570 children born after non-ART (3.9‰) were diagnosed with DM1. No clear differences in the odds of DM1 were found when comparing children born after FET and fresh ET (aOR 0.79, 95% CI: 0.41–1.53) or FET and non-ART (aOR 0.74, 95% CI: 0.40–1.38). Finally, no difference in the risk of DM1 was found in comparisons of children born after fresh ET and non-ART children (aOR 1.01, 95% CI: 0.84–1.23) (Table 3).

No differences were observed when comparing odds of DM1 in children born after fresh ICSI versus fresh IVF (aOR 1.12, 95% CI: 0.72–1.73). No analyses were performed for frozen ICSI versus frozen IVF owing to a limited number of cases in both groups.

Adjustment for LGA or birthweight (continuous and categorical) did not change the result.

### Subgroup analyses

There were no clear differences in associations according to country or sex, and restriction to children born at term, children born with birthweight within the normal range, multiples, or children with a follow-up of at least 2 years did not change the results (Supplementary Table S3).

Restricting the definition of maternal DM1 to pre-gestational diagnoses did not change the results.

## Discussion

In this Nordic cohort study, we found no difference in the risk of DM1 between the ART and non-ART groups, though in the youngest birth cohort, the risk was higher for ART-conceived singletons. We found no difference in the risk of DM1 in singletons born after IVF, ICSI, or FET.

### Comparisons to other studies

The literature on associations between prenatal and perinatal characteristics and the risk of DM1 is comprehensive, with firm evidence of an increased risk of DM1 in children born preterm, LGA or with high birthweight (≥4000 g) (Haynes *et al.*, 2007; Harder *et al.*, 2009; Cardwell *et al.*, 2010; Metsälä *et al.*, 2020; Magnusson *et al.*, 2021), while fewer studies have compared the risk of DM1 in children born from ART and non-ART conception.

In coherence with our results, Norrman *et al.* (2020) found no increased risk of DM1 in children born after ART compared to non-ART, with an adjusted HR of 1.06 (95% CI: 0.92–1.22), comparing 47 938 ART with 3 090 602 non-ART singletons in Sweden. Contrary to our findings, they found a higher risk of DM1 in children born after FET compared with fresh ET and natural conception. We identified only 10 DM1 cases in the FET group (n = 7985), while there were 45 children with DM1 in the FET group (n = 11 200) in the Swedish study, and we cannot exclude that the differences in association could be caused by random variation. FET is associated with a higher risk of being born LGA and with high birthweight (Shih *et al.*, 2008; Berntsen and Pinborg, 2018; Maheshwari *et al.*, 2018). Norrman did not adjust for birthweight or LGA in this analysis, which may have mediated an increase in the risk of DM1 in the FET population (Norrman *et al.*, 2020).

In a previous cohort study in Denmark overlapping with the oldest cohorts in our study (1995–2003), including 565 116 singletons with 14 985 born after ART, Kettner *et al.* (2016) found no difference in the risk of DM1. They did, however, find an increased risk of DM1 in children born after ovulation induction or IUI (n = 2011), during which the mother was exposed to FSH, compared to non-ART (HR 3.22, 95% CI: 1.20 to 8.64). Kettner *et al.* (2016) suggested that the association between gonadotrophin treatment and DM1 in the offspring could be a result of confounding by indication, arguing that women treated with gonadotrophins in IUI might share characteristics related to an increased risk of DM1 in their offspring. We hypothesize that these factors may be PCOS or overweight, as low-dose FSH stimulation is prescribed for mono-ovulation prior to IUI in women with anovulation (Homburg, 2003). However, Kettner *et al.* (2016) did not account for PCOS, anovulation, or BMI in their analyses. Further, high doses of gonadotrophin are normally a part of IVF and ICSI treatments, where no increased risk was found (Kettner *et al.*, 2016). A distinction between IUI, IVF, and ICSI, and children born without the use of ART in our cohort would be of interest, owing to the difference in patient characteristics. Unfortunately, we were not able to perform analyses on IUI treatments as these were included in the population considered non-ART in the CoNARTaS cohort. As the children conceived by IUI comprise a very low proportion of the full non-ART population, we do not expect a potential higher risk of DM1 in this subpopulation to bias our results. Hargreave *et al.* (2016) also investigated the risk of DM1 in children born in Denmark, comparing 110 393 children of mothers with a diagnosis of infertility and an unexposed group of 1 550 519 children born between 1987 and 2010, also overlapping with our cohort. Although it was not specified whether the children were conceived by ART or non-ART, the results were reassuring as they found no increased risk of DM1 in children of women who had experienced infertility (Hargreave *et al.*, 2016). A study by Klemetti *et al.* (2006) investigated the health of ART-conceived children up to 4 years of age including 4559 ART and 190 398 non-ART children in Finland. While perinatal outcomes and hospital episodes were more common in the children conceived by ART, they did not have significantly higher ORs for DM1 compared to non-ART children (Klemetti *et al.*, 2006). Finally, a second Finnish study by Terho *et al.* (2022) looked at the health of singletons conceived by FET by comparing the risk of diseases grouped by chapters of ICD-10 codes. They included 1825 FET, 2933 fresh ET and 31 136 non-ART children and found no increase in adjusted HRs of endocrine, nutritional, and metabolic diseases (ICD-10-chapter E), between all three groups (Terho *et al.*, 2022). However, both studies found a slightly higher prevalence of DM1 in the ART group, and endocrine, nutritional, and metabolic diseases in the FET and fresh ET groups compared to the non-ART children, respectively. Both the Danish and Finnish studies mentioned above overlap with our cohort.

Maternal diabetes was the covariate most strongly associated with risk of DM1 in the offspring. Female sex and maternal smoking during pregnancy were both associated with lower risk. It is well known that male sex is a risk factor for DM1 (Karvonen, 2006). Magnus *et al.* (2018) suggested that the impact of smoking on the risk of DM1 in offspring may be mediated through reduced birthweight among the offspring of smoking mothers. However, upon adjusting for covariates, including birthweight, an inverse association between maternal smoking and the risk of DM1 in the offspring was observed (Magnus *et al.*, 2018).

### Strengths and limitations

We had access to a large, full-population cohort from three countries with detailed register-based information, allowing examination of potential associations with specific subtypes of ART. Although the median age at the end of follow-up in this study was 8.3 years in the ART group and thus appropriate to assess the risk of early-onset DM1, the main limitation of the study is the relatively short follow-up. The incidence rate of DM1 peaks during ages 10–14 years (Maahs *et al.*, 2010), hence a longer follow-up would benefit all analyses and specifically the subgroup analyses.

The follow-up time in this study was shorter for the children from the most recent birth cohorts, with ART-conceived children accounting for an increasing proportion of the birth cohorts over the latest decades. The higher risk of DM1 in the ART population in the youngest age group demands further research, when long-term data become available in this group. With only 19 ART-conceived children being diagnosed with DM1 in the youngest age group, we cannot rule out random variation as a possible explanation. No consistent differences in the age at diagnosis or age at end of follow-up between the ART- and non-ART-conceived children were found in the sample where age at onset could be reliably estimated from date of first diagnosis (Supplementary Fig. S2) or within the birth year categories (Supplementary Table S1). To overcome the challenge of differences in follow-up, we performed both multivariable logistic regression analyses stratified by year of birth categories with adjustment for year of birth (Table 2), and Cox regression analyses in the sample where age at onset could be reliably estimated from date of first diagnosis (Supplementary Table S2).

The validity of our analyses relies on the correct registration of DM1 diagnoses, which has been validated in the Danish NPR (Svensson *et al.*, 2007). Even though registration errors will always exist (Sund *et al.*, 2010), we do not expect such misclassification to be differential between ART and non-ART. In the validation study by Svensson *et al.* (2007), only 3.5% of registered DM1 diagnoses were incorrect when using the E10 code in the Danish NPR (Svensson *et al.*, 2007). In Norway, where NPR data were available from 2008, we expect that DM1 patients with an onset before 2008 would still be registered with a DM1 diagnosis after 2008, owing to recommendations of regular control visits in specialized health care. Further, children with DM1 are hospitalized shortly after disease onset and first diagnosis. Hence, children with unregistered DM1 would be unlikely and, more importantly, we do not expect registration differences between the ART and non-ART groups.

A small fraction of the Finnish mothers (13.5%) and children (1.5%) registered as having DM1 in this study were registered in an ICD-8 or ICD-9 classification, with no differentiation between DM1 or Type 2 diabetes. This is particularly relevant when adjusting for maternal diabetes since the risk of DM1 in the offspring is relatively higher when the mother has DM1 compared to Type 2 diabetes (Hussen *et al.*, 2015). We do expect a difference in the DM1 registration between the mothers of ART- and non-ART-conceived children, since the non-ART group is proportionally larger in the early birth years, where diagnoses were classified by ICD-8 or ICD-9 codes. In our main analyses, we defined maternal DM1 as a diagnosis at any time during follow-up because it presumably captures genetic predisposition better than pre-gestational DM1. Restricting maternal DM1 to pre-gestational DM1 would have led to less collider bias from conditioning on the future, but potentially more residual confounding. Additionally, ART-conceiving women were older, increasing their likelihood of being diagnosed with DM1 prior to pregnancy, even if their underlying age-specific risk was similar to the non-ART group.

The lack of information on paternal DM1 could lead to residual confounding because we expect the proportion of paternal DM1 to be higher in the ART group since DM1 affects male fertility (Holstein *et al.*, 2012; Wiebe *et al.*, 2014; la Vignera *et al.*, 2015). Further, paternal DM1 is a stronger predictor for DM1 compared to maternal DM1 (Hämäläinen and Knip, 2002).

Moreover, we had no information on parental country of origin, which is a limitation considering the differences in the risk of DM1 associated with ethnicity (Maahs *et al.*, 2010).

## Conclusion

In this large Nordic cohort, risk of DM1 was similar for children conceived with and without ART, except in the youngest cohort, where there was a higher risk among ART-conceived based on a very limited number of events. There were no clear differences in risk according to ART method (IVF, ICSI, or FET specifically). However, the number of DM1 diagnoses among children born after FET and in the youngest age group were limited, and continued surveillance of the risk of DM1 is needed. Overall, our findings are reassuring, especially considering the concomitantly increasing number of children born from ART and number of children diagnosed with DM1 globally.

## Supplementary Material

hoae021_Supplementary_Figure_S1

hoae021_Supplementary_Figure_S2

hoae021_Supplementary_Tables

## Data Availability

The data underlying this study cannot be shared publicly because the data sources are national register information which have been made available for this specific study. Thus, CoNARTaS data were analysed within the Statistics Denmark research server. The use of microdata from the national registers accessed from Statistics Denmark follows the General Data Protection Regulation. Due to data protection and privacy legislations, no data are available.
