# Supplementary figures and images for: The risk of Type 1 diabetes in children born after ART: a Nordic cohort study from the CoNARTaS group
Source: Hum Reprod Open. 2024 Apr 10;2024(2):hoae021. doi: 10.1093/hropen/hoae021 (PMC11061545; doi:10.1093/hropen/hoae021)

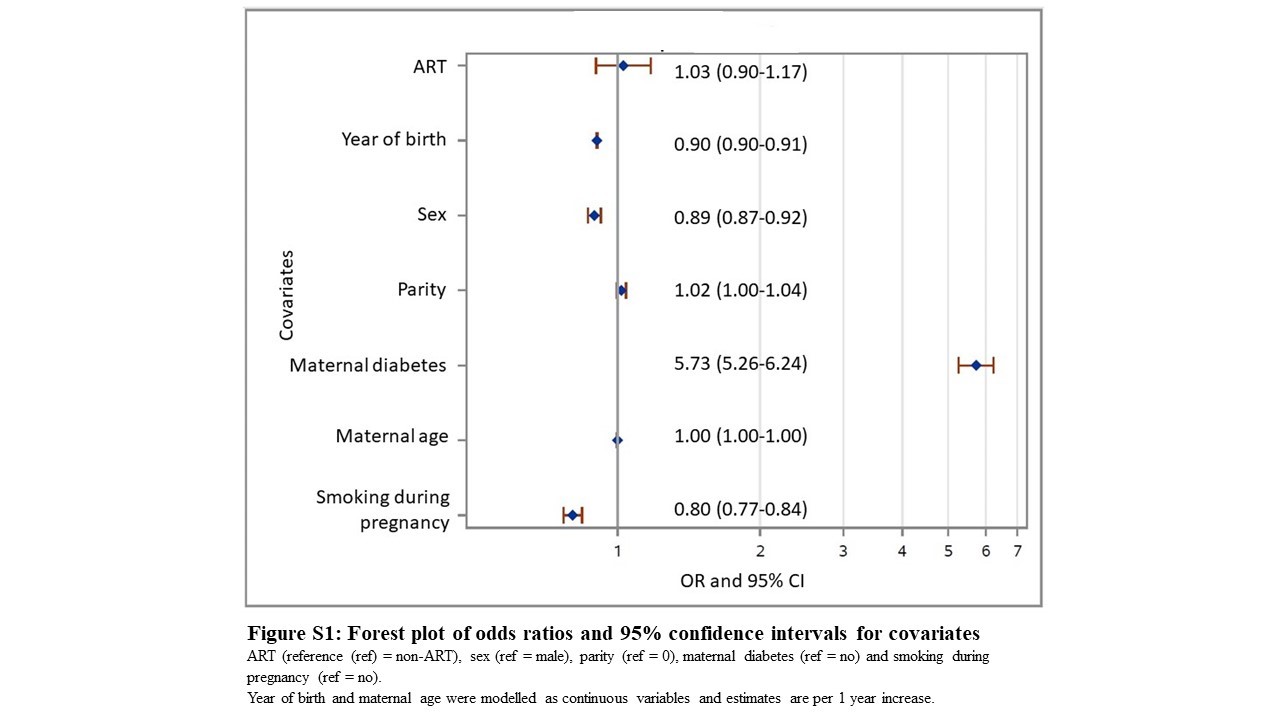

Supplement: hoae021_Supplementary_Figure_S1 [file hoae021_supplementary_figure_s1.jpeg]

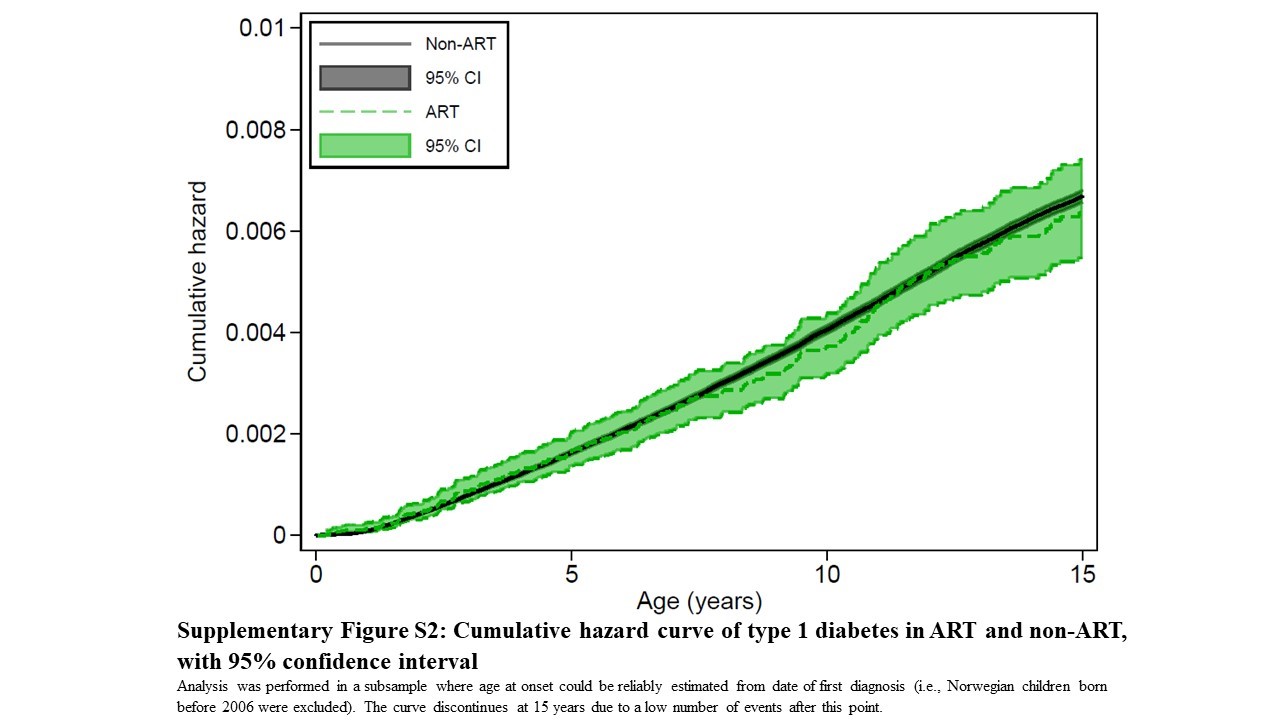

Supplement: hoae021_Supplementary_Figure_S2 [file hoae021_supplementary_figure_s2.jpeg]
