# Supplementary material for: The risk of Type 1 diabetes in children born after ART: a Nordic cohort study from the CoNARTaS group
Source: Hum Reprod Open. 2024 Apr 10;2024(2):hoae021. doi: 10.1093/hropen/hoae021 (PMC11061545; doi:10.1093/hropen/hoae021)
Supplement: hoae021_Supplementary_Tables [file hoae021_supplementary_tables.docx]

**Supplementary Table S1:** Follow-up and age at first diagnosis of type 1 diabetes for singletons born after ART and non-ART in different time periods: sample restricted to Denmark 1994–2014, Finland 1990–2014, and Norway 2006–2015.

|  | **Follow-up (years)** | | **Age at first type 1 diabetes diagnosis (years)** | |
| --- | --- | --- | --- | --- |
| **Birth year** | **ART**  **N = 68 004**  **median (IQR)** | **Non-ART**  **N = 3 208 333**  **median (IQR)** | **ART**  **N = 68 004**  **median (IQR)** | **Non-ART**  **N = 3 208 333**  **median (IQR)** |
| 1991–1995⁑ | 21.1 (20.4 – 22.3) | 21.7 (20.8 – 23.3) | 11.9 (9.3 – 13.7) | 10.6 (6.4 – 14.2) |
| 1996–2000 | 17.2 (16.0 – 18.4) | 17.5 (16.2 – 18.8) | 8.7 (4.9 – 11.4) | 9.1 (5.4 – 12.3) |
| 2001–2005 | 12.4 (11.1 – 13.6) | 12.4 (11.2 – 13.7) | 7.3 (4.3 – 10.4) | 6.4 (3.7 – 9.2) |
| 2006–2010 | 7.3 (6.1 – 8.5) | 7.5 (6.2 – 8.7) | 3.9 (2.7 – 5.5) | 4.1 (2.5 – 5.8) |
| 2011–2015⁑ | 2.8 (1.6 – 3.9) | 2.8 (1.7 – 3.9) | 1.7 (1.2 – 2.4) | 1.9 (1.3 – 2.7) |
| Total | 7.4 (3.9 – 12.6) | 10.2 (5.5 – 17.4) | 6.1 (3.0 – 10.3) | 7.5 (4.1 – 11.4) |

IQR interquartile range.

⁑ For the birth year of 1990-1993 only data from Finland were included. No ART children born in 1990 were diagnosed with type 1 diabetes during follow-up. For the birth year of 2015 only data from Norway were included.

**Supplementary Table S2:** Sensitivity analysis comparing logistic regression to Cox regression in children with valid time of diagnosis.

|  | **Type 1 diabetes – no. (‰)** | **Median time of follow-up ⎯ years (IQR)** |
| --- | --- | --- |
| **ART** | 220/68 004 (3.2) | 7.4 (3.9 – 12.6) |
| **Non-ART** | 14 339/3 208 333 (4.5) | 10.2 (5.5 – 17.4) |
| **Adjusted OR (95% CI)^1^** | **Adjusted OR (95% CI)^2^** | **Adjusted OR (95% CI)^3^** |
| 1.04 (0.91 – 1.19) | 1.04 (0.91 – 1.19) | 1.04 (0.91 – 1.20) |
| **Adjusted HR (95% CI)^1^** | **Adjusted HR (95% CI)^2^** | **Adjusted HR (95% CI)^3^** |
| 1.01 (0.89 – 1.16) | 1.02 (0.896 – 1.17) | 1.03 (0.89 – 1.18) |

Associations are presented as adjusted odds ratios (OR) and hazard ratios (HR).

IQR interquartile range.

1: adjusted for year of birth

2: adjusted for year of birth, sex of the child, parity, maternal age, and maternal diabetes

3: adjusted for year of birth, sex of the child, parity, maternal age, maternal diabetes, and maternal smoking during pregnancy

**Supplementary Table S3:** Multivariable logistic regression analyses for singletons born after ART versus non-ART by country, born at term, normal birthweight (2500 – 4000g), sex, children with a minimum of 2 years of follow-up and multiples – risk of type 1 diabetes during follow-up.

|  |  | **Type 1 diabetes**  **no. (‰)** | **Median follow-up ⎯ yrs (IQR)** | **Adj. OR^1^**  **(95% CI)** | **Adj. OR^2^**  **(95% CI)** | **Adj. OR^3^**  **(95% CI)** |
| --- | --- | --- | --- | --- | --- | --- |
| **Denmark** | **ART** | 62/30 368 (2.0) | 8.5 (4.6 – 13.4) | 1.08  (0.84 – 1.39) | 1.10  (0.85 – 1.42) | 1.15  (0.88 – 1.50) |
|  | **Non-ART** | 3 226/1 224 803 (2.6) | 11.6 (6.4 – 16.9) |  |  |  |
| **Finland** | **ART** | 129/21 952 (5.9) | 9.6 (4.9 – 15.6) | 1.04  (0.87 – 1.24) | 1.00  (0.84 – 1.20) | 1.00  (0.84 – 1.19) |
|  | **Non-ART** | 10 380/1 423 900 (7.3) | 13.8 (7.3 – 20.3) |  |  |  |
| **Norway** | **ART** | 68/23 864 (2.8) | 7.1 (3.4 – 12.5) | 1.01  (0.79 – 1.28) | 1.01  (0.79 – 1.29) | 1.20  (0.89 – 1.63) |
|  | **Non-ART** | 8 347/1 754 716 (4.8) | 16.0 (7.7 – 23.7) |  |  |  |
| **Term** | **ART** | 235/69 993 (3.4) | 8.2 (4.2 – 13.8) | 0.99  (0.87 – 1.13) | 0.98  (0.86 – 1.12) | 1.02  (0.89 – 1.18) |
|  | **Non-ART** | 20 236/4 111 568 (4.9) | 13.6 (7.0 – 20.2) |  |  |  |
| **Normal**  **BW** | **ART** | 209/59 872 (3.5) | 7.1 (3.4 – 12.5) | 1.04  (0.91 – 1.19) | 1.01  (0.88 – 1.16) | 1.07  (0.92 – 1.23) |
|  | **Non-ART** | 16 563/3 406 412 (4.9) | 16.0 (7.7 – 23.7) |  |  |  |
| **Female sex** | **ART** | 102/37 269 (2.7) | 8.2 (4.3 – 13.8) | 0.84  (0.69 – 1.03) | 0.84  (0.69 – 1.03) | 0.89  (0.73 – 1.10) |
|  | **Non-ART** | 9 988/2 146 158 (4.7) | 13.7 (7.2 – 20.3) |  |  |  |
| **Male sex** | **ART** | 157/38 915 (4.0) | 8.4 (4.3 – 14.0) | 1.11  (0.95 – 1.31) | 1.09  (0.93 – 1.28) | 1.14  (0.96 – 1.36) |
|  | **Non-ART** | 11 962/2 257 261 (5.3) | 14.0 (7.2 – 20.3) |  |  |  |
| **Follow-up ≥2 years** | **ART** | 257/68 915 (4.0) | 9.2 (5.4 – 14.5) | 0.98  (0.87 – 1.11) | 0.97  (0.86 – 1.10) | 1.02  (0.89 – 1.17) |
|  | **Non-ART** | 21 935/4 187 924 (5.3) | 14.4 (8.1 – 20.5) |  |  |  |
| **Multiples** | **ART** | 134/31 420 (4.3) | 11.9 (6.7 – 16.5) | 1.18  (0.97 – 1.43) | 1.21  (0.98 – 1.50) | 1.17  (0.93 – 1.48) |
|  | **Non-ART** | 509/119 246 (4.3) | 14.4 (6.9 – 19.5) |  |  |  |

Associations are presented as adjusted ORs.

Adj. adjusted, OR: odds ratio, IQR interquartile range, yrs years.

1: adjusted for year of birth

2: adjusted for year of birth, sex of the child, parity, maternal age, and maternal diabetes

3: adjusted for year of birth, sex of the child, parity, maternal age, maternal diabetes, and maternal smoking during pregnancy
